# The Role of Formulations in the Ageing Process of Vinyl Acetate Based Emulsion Films: A Multivariate Approach

**DOI:** 10.3390/polym16192841

**Published:** 2024-10-08

**Authors:** Carolina Viana, Karin Wieland, Susana França de Sá, Eva Mariasole Angelin, Valentina Pintus, Joana Lia Ferreira

**Affiliations:** 1 LAQV-REQUIMTE, Department of Conservation and Restoration, NOVA School of Science and Technology, Universidade NOVA de Lisboa, 2829-516 Caparica, Portugal; c.viana@campus.fct.unl.pt (C.V.); susana.sa@fct.unl.pt (S.F.d.S.); 2 Competence Center CHASE GmbH, Ghegastrasse 3, A-1030 Vienna, Austria; 3 Conservation-Restoration, Art Technology and Conservation Science, Technical University of Munich, Oettingenstr. 15, 80538 Munich, Germany; eva.angelin@tum.de; 4 Institute of Science and Technology in Art, Academy of Fine Arts, Schillerplatz 3, A-1010 Vienna, Austria; v.pintus@akbild.ac.at; 5 Institute for Conservation-Restoration, Modern-Contemporary Art, Academy of Fine Arts Vienna, Schillerplatz 3, A-1010 Vienna, Austria; 6 CIUHCT—Interuniversity Center for the History of Sciences and Technology, Department of Conservation and Restoration, NOVA School of Science and Technology, Universidade NOVA de Lisboa, 2829-516 Caparica, Portugal

**Keywords:** poly(vinyl acetate), aqueous vinyl emulsions, principal component analysis, hierarchical cluster analysis, multivariate analysis, chemometrics

## Abstract

Vinyl acetate (VAc)-based emulsions represent one of the main media used by modern and contemporary artists. Their long-term behaviour is still not completely understood, especially due to the scarce knowledge on the influence of other compounds in the formulation, which may impact ageing over time. Besides the polymer backbone based on vinyl acetate, other co-monomers and additives can be added to the emulsion to alter the final film’s physical, chemical, and optical properties. By extension, the formulation will also impact the long-term stability of artworks and objects on which it has been applied, as well as possible current and future conservation interventions such as cleaning. For those reasons, studies shedding light on the correlation between composition and long-term stability are largely necessary. In this study, different emulsions, including homopolymers, copolymers, plasticised, and un-plasticised compositions, were gathered and artificially aged. A multivariate analyses approach based on the application of principal component analyses (PCA) and hierarchical cluster analyses (HCA) was employed for the first time on the combination of data obtained by pH, contact angle (CA), colour measurements, Fourier transform infrared spectroscopy in attenuated total reflection (FTIR-ATR), and size exclusion chromatography (SEC). This approach helped to highlight the changes that occurred during ageing and find correlations with the formulation compositions. The results further sustain the thesis that not all vinyl acetate-based emulsions are chemically the same and that their formulation deeply impacts their long-term behaviour.

## 1. Introduction

Vinyl acetate (VAc)-based emulsions have been used as binders in paintings at least since the 1960s by modern and contemporary artists [1]. These emulsions offered new characteristics such as fast drying, a lower tendency towards yellowing, and colour diversity, plus a lower price, which made them attractive to artists [2]. VAc-based emulsions can also have complex formulations since a wide range of additives might be incorporated to change their optical, physical, and chemical properties [3]. Each additive has its own stability and might influence the deterioration rate of the resulting film [4,5]. Moreover, emulsion formulations have changed throughout the years to comply with green laws and new customer requirements. This new information is often patented and not fully disclosed, which contributes to the difficulty of knowing their detailed chemical composition [2,6].

Physicochemical methods are often used in combination with artificial ageing to characterise and understand the ageing behaviour of polymers [7,8,9,10,11,12]. However, in comparison with acrylic painting media and adhesives, VAc-based formulations have been less studied. The most frequently employed techniques for their characterisation are Fourier transform infrared (FTIR) and Raman spectroscopies, atomic force microscopy (AFM), nuclear magnetic resonance (NMR), scanning electron microscopy with dispersive X-ray analysis (SEM/EDX), thermogravimetric analysis (TGA), differential scanning microscopy (DSC) and pyrolysis—gas chromatography/mass spectrometry (Py-GC/MS) [13,14]. Out of these, FTIR and Py-GC/MS are the most frequently employed [14].

These techniques usually produce large amounts of data, which may be difficult to interpret and visualise, especially when a multi-sensor approach is pursued. To this end, unsupervised chemometric algorithms, such as principal component analyses (PCA) and hierarchical cluster analyses (HCA), can be applied to break down the complexity of the dataset and facilitate data interpretation [15,16,17,18,19,20,21]. PCA is a multivariate method that enables the reduction in the dimensionality of large datasets by a linear transformation of the data space, creating a new coordinate system of uncorrelated variables guided by the directions of the greatest variance in the original dataset [22,23]. HCA is a classification algorithm based on the assumption that similar datapoints are close together in the *n*-dimensional dataspace. The result is depicted in a dendrogram where the branch length indirectly correlates with the similarity between clusters [24,25].

Chemometric tools have already been applied for the characterisation of different materials in the field of cultural heritage [25,26]. PCA has been used for the classification and identification of binding media and their components [18,27,28,29,30,31,32], to study paint stratigraphy [17,33], and to predict ageing behaviours [15,30,32]. Specifically for modern art paint materials, PCA has been used to characterise, identify, and group different polymeric binders [16], including vinyl-based varnishes [34], and to investigate their chemical stability and ageing behaviour [20,35,36], whereas HCA has found fewer applications in cultural heritage [25,37] being more often used in combination with other multivariate analysis methods to validate results [18,20,24,26,28].

This research aims to understand the impact that additives have in different formulations on the long-term stability of vinyl emulsion paints. Therefore, selected emulsions were exposed to artificial light ageing and characterised following a multi-analytical approach (pH, contact angle (CA), and colour measurements, FTIR-ATR, and SEC. Obtained data were then analysed in a combined multivariate approach using unsupervised classification algorithms principal component analyses and hierarchical cluster analyses. For the first time, several characterisation techniques were combined in a single data matrix and analysed simultaneously via a multivariate analysis to better highlight and formulate possible correlations such as ageing trends and cluster emulsions according to formulation type.

## 2. Experimental

### 2.1. Materials and Methods

In this work, the ageing of seven different formulations was investigated. The composition of the formulations was characterised by infrared spectroscopy and Py-GC/MS presented in our previous work [6]. As reported in Table 1, six VAc-based emulsions were acquired from Portuguese fine arts stores and chemical industries. Also, pure PVAc homopolymer in the form of beads with M_W_ ≈ 83,000 was purchased from Sigma Aldrich and served as a control sample after having been prepared as film (pure PVAc was dissolved in acetone (reagent grade material) in a 20/80 (*w*/*v*) proportion before application on a glass slide). The remaining emulsions were cast on glass slides with a film applicator and had an average film thickness of 90 μm. Film thickness was measured using a digital calliper with a linear capacitive system by Powerfix Profi (Milomex, Somerset, UK) with a ±0.01 mm accuracy. All films were left to dry for three weeks (until no weight variation was detected) and placed in an artificial light ageing chamber. A total of 42 films (6 replicas for each formulation, 7 different formulations) were irradiated for 0, 500, 1000, 2000, and 4000 h, and their physical and chemical changes were characterised at each irradiation dosage through pH, CA, colour measurements, FTIR-ATR, and SEC. Each film was analysed three times in three different areas to ensure data representativeness.

### 2.2. Instrumentation

The light ageing experiment was carried out using a CO.FO.ME.GRA 3000 light-chamber equipped with a Xenon-arc light source (λ ≥ 300 nm), with constant irradiation of 800 W/m^2^ and black standard temperature (BST) of 50 °C, to simulate natural ageing conditions in shorter times. The total amount of irradiance at the end of the experiment was 13762 MJ/m^2^. pH measurements were carried out with a pH Check digital pH metre with ATC Water series 5040-0303 by TFA Dostmann (TFA Dostmann GmbH & Co. KG, Wertheim, Germany) (±0.01 accuracy). Water contact angles (degrees) of the films were measured at room temperature with distilled water droplets using an optical contact angle measurement and contour analysis systems of the OCA 15EC series from Dataphysics (DataPhysics Instruments GmbH, Filderstadt, Germany). Colour measurements were performed using a Konica Minolta CR−410 Colourimeter (Konica Minolta, Inc., Lisbon, Portugal). The optical system of the measuring head uses diffuse illumination from a pulsed Xenon lamp over a measuring area of 50 mm in diameter, 10° viewing angle geometry and D65 illuminant. Measurements were recorded on the SpectraMagic NX software v3.40. Calibration was performed with bright white and black standard plates before each measurement. The average and standard deviation values for each L*, a* and b* coordinates were calculated as well as the total colour variation (∆E*) according to the CIE 2000 formula in the Techkon ∆E* Calculator. To follow yellowing, the b* value (ranging from negative values (blue) to positive (yellow)) [38] was specifically selected for further analysis in this study.

FTIR-ATR spectra were collected with the handheld Agilent 4300 spectrophotometer (Agilent, Santa Clara, CA, USA), equipped with a ZnSe beam splitter, a Michelson interferometer, and a thermoelectrically cooled DTGS detector. Spectra between 4000 and 650 cm^−1^ were acquired with a diamond ATR module, 128 scans and 4 cm^−1^ resolution. Background spectra (air) were collected before every acquisition.

Molecular weight distributions were determined with a size exclusion chromatography (SEC) apparatus (Knauer Smartline) (Separations Analytical Instruments, Hendrik-Ido-Ambacht, The Netherlands). Separation was performed with a Phenomenex Phenogel Linear LC Column 300 × 7.8 mm. The operating temperature was 25 °C, using chloroform as an eluent with a flow rate of 0.5 mL/min. The injection volume was 50 μL. Samples were dissolved in chloroform in a concentration range of 0.1–0.2%. Calibration was conducted with polystyrene standards −12 points, range 2520 kDa–0.37 kDa (Supelco). The Mark-Houwink constants at 30 °C are PS/CHCl_3_ k = 11.2/α = 0.73|PHB/CHCl_3_ k = 20.3/α = 0.72. Material for analysis was collected from the surface of the VAc-based films using a scalpel. The injections were achieved in duplicate. The software for data collection and treatment was Clarity v5.0.5.98.

### 2.3. Data Pre-Processing and Multivariate Statistical Analysis

Data pre-processing, analysis and visualisation were performed in Python 3.9 using Pandas v1.5.3 [39], sklearn v1.3.0 [40], SciPy v1.10.1 [41], NumPy v1.24.3 [42], and matplotlib v3.7.1 [43] packages.

**Figure 1:**
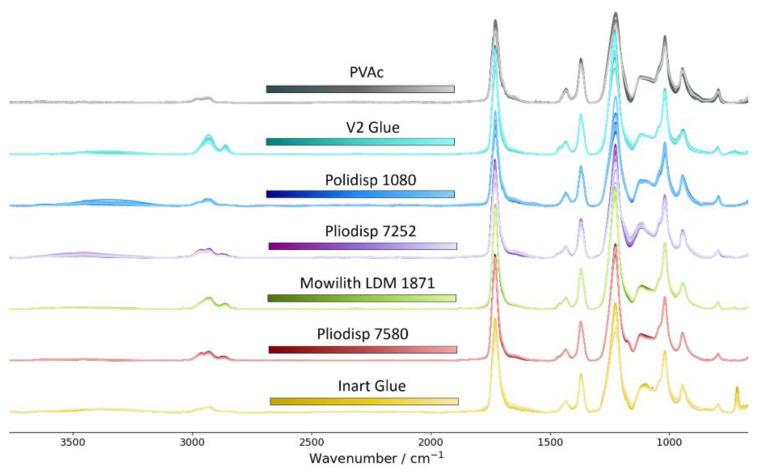
FTIR-ATR spectra (unaged and aged) of PVAc (grey), V2 Glue (turquoise), Polidisp 1080 (blue), Pliodisp 7525 (purple), Mowilith LDM 1871 (green), Pliodisp 7580 (red), and Inart Glue (yellow) after baseline correction and normalisation. An offset between spectra was added for better visualisation. Ageing time is indicated by colour brightening (lighter corresponds to longer ageing time).

FTIR-ATR spectra of each sample (unaged and aged) were collected in triplicates corresponding to three different measurement spots on the sample surface. For better visualisation and comparability, the spectra (unaged and aged) were baseline corrected using asymmetric least squares smoothing by Eilers and Boelens (λ = 10^5^, *p* = 0.0001, 10 iterations) [44] and normalised to area = 1 (Figure 1).

**Figure 2:**
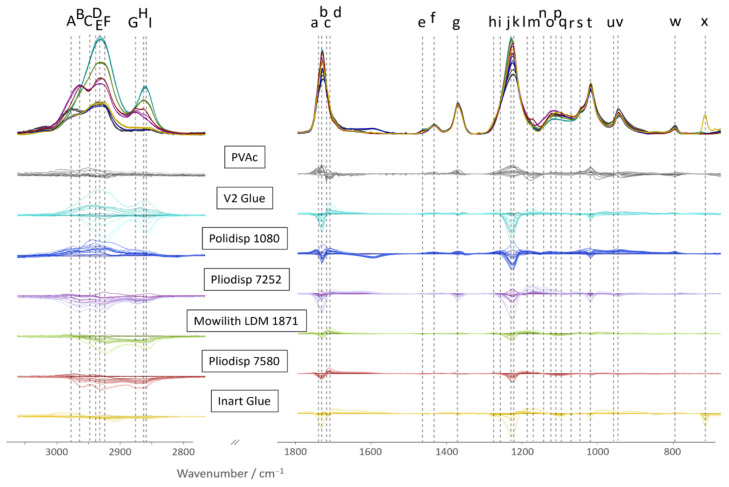
Top row (no ageing, all formulations at t = 0 h): CH stretching and fingerprint region of FTIR-ATR spectra (baseline corrected and normalised to area = 1) of the different VAc-based emulsions. Remaining seven rows: difference spectra relative to t = 0 h for each formulation at different exposure times highlight the spectral differences induced by light exposure. Ageing time is indicated by colour brightening (lighter corresponds to longer ageing time). The intensity values in the CH stretching region (2750–3800 cm^−1^) were multiplied by a factor of 8 for better visualisation. The letters used as labels are listed in Table 2.

To reduce the dimensionality of the dataset, specific wavenumbers were carefully selected for the data analysis. On the one hand, this enables the data analysis to have a set focus on specific predefined areas of interest. On the other hand, this also entails the risk of accidentally excluding crucial information. Detailed sample knowledge is required for defining this list of wavenumbers to be included in the multivariate statistical analysis (Table 2). Bands from the fingerprint and CH-stretching regions of the spectra recorded at t = 0 h were included, as well as spectral changes observed in the difference spectra at the respective ageing times (Figure 2).

The baseline-corrected intensity values of the selected list of wavenumbers were merged with the information obtained from additional variables describing sample properties such as pH, CA and colour (b* value) measurements, molecular weight (M_W_), and polydispersity (PD). Difference values to the sample at t = 0 h were calculated to set the focus of the analysis on the chemical changes occurring during ageing rather than differences between formulations. While for FTIR-ATR spectra, more data treatment was required due to the amount of information (>800 datapoints/spectrum) detected in a single measurement. For all the latter variables mentioned, the calculation of difference values was considered sufficient.

Finally, the combined data matrix was standardised (µ = 0, σ = ±1) along each variable prior to performing a PCA of the multi-sensor dataset. This standardisation step was key, as different analytical techniques result in values of different size ranges. Due to data standardisation, the light-induced changes in the dataset are highlighted and also scaled through division by the standard deviation of each variable. PCA is a linear transformation of the highly dimensional data space to achieve a down-projection to a 2- or 3-dimensional space. The idea is that the transformation highlights important features explaining most of the data variance in the original coordinate system. The combined analysis of data comprising several different analytical methods allows one to highlight certain correlations during the ageing process across the investigated formulations. To illustrate similarities in the samples based on the selected variables across the employed analytical methods, HCA of the scores of the first five principal components (PCs), explaining ≈85% of the total variance, was performed. Samples that cluster in the same (sub-)branches of the resulting dendrogram are similar, i.e., show similar ageing behaviour.

## 3. Results and Discussion

**Figure 3:**
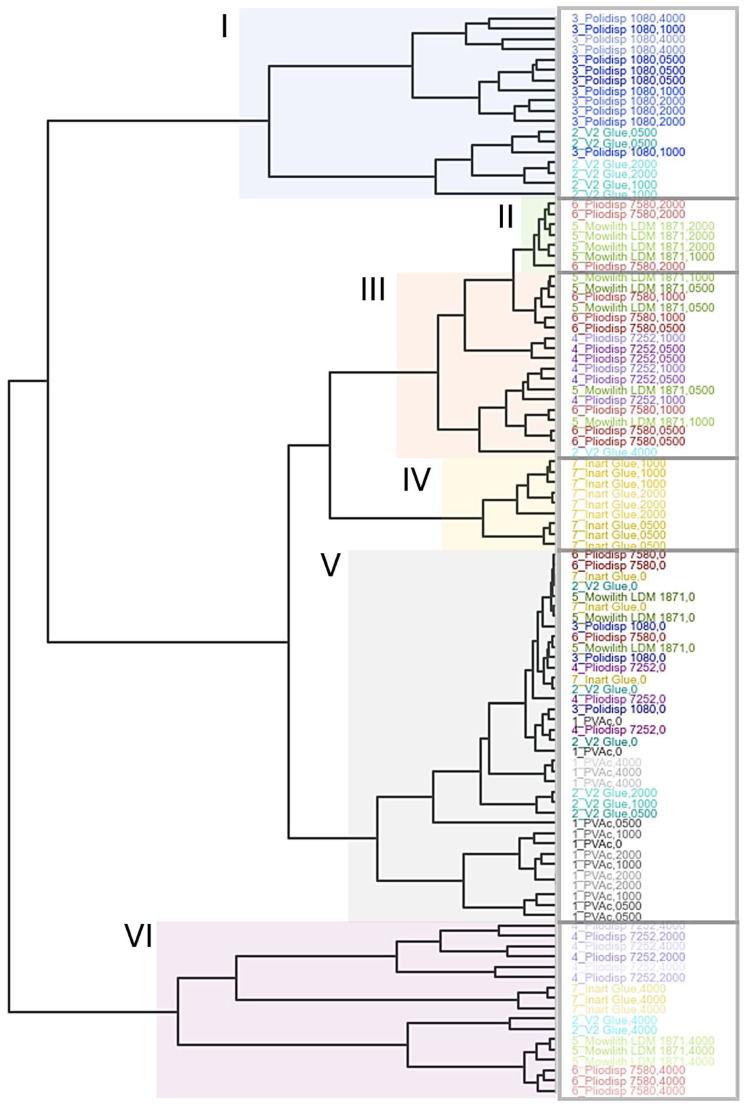
Dendrogram of the HCA of the PC1, PC2, PC3, PC4, and PC5 scores with samples coloured according to formulation type (PVAc—grey, V2 Glue—turquoise, Polidisp 1080—blue, Pliodisp 7252—purple, Mowilith LDM 1871—green, Pliodisp 7580—red and Inart Glue—yellow) and ageing time (higher transparency indicates longer ageing time). Clusters in the dendrogram are highlighted according to ageing differences. Cluster I contains mostly Polidisp 1080 and V2 Glue, Cluster II contains samples at t = 2000 h, Cluster III at t = 500–1000 h, Cluster IV is only made of Inart Glue, Cluster V contains unaged samples of all formulations and aged PVAc, and Cluster VI is mostly samples at t = 4000 h.

**Figure 4:**
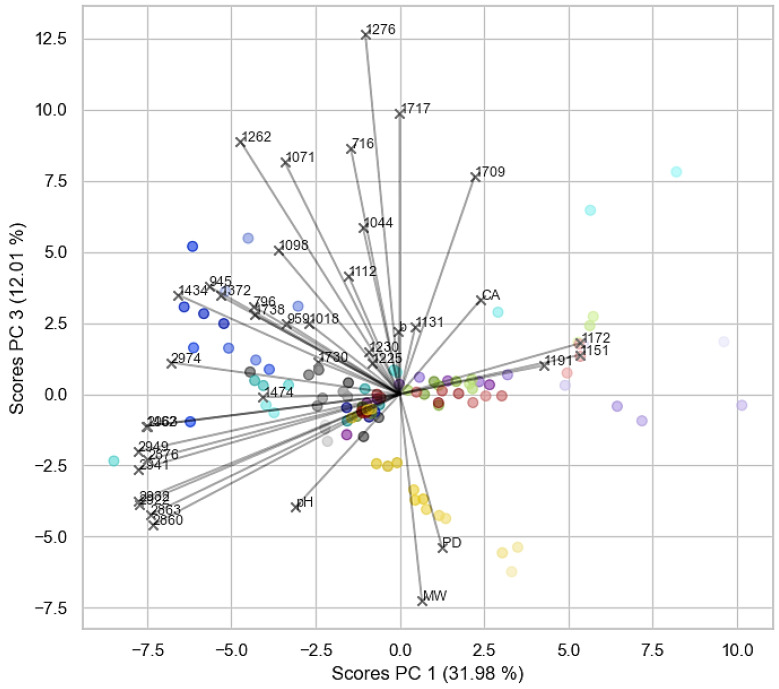
Bi-plot depicting the scores and loadings of PC1 vs. PC3. The colour of the scores corresponds to the formulation type: PVAc (grey), V2 Glue (light blue), Polidisp 1080 (dark blue), Pliodisp 7252 (purple), Mowilith LDM 1871 (green), Pliodisp 7580 (red) and Inart Glue (yellow), and its transparency to the ageing time (higher transparency indicates longer ageing time).

The main results of this study are shown here in a dendrogram and a bi-plot. An overall description of these findings will be presented hereafter. A more thorough analysis of the results will then be discussed in the subsequent paragraphs for each formulation type. Figure 3 shows the dendrogram for scores of PCs, 1–5 explaining 85% of the total data variance. Important correlations and the impact of different variables on the clustering are subsequently highlighted in the corresponding 2-dimensional bi-plot (Figure 4) showing a good separation of the differently aged samples. All score plots and loadings are depicted in the Supplementary Materials (Figures S1 and S2).

In Figure 3, the outermost end of each sub-branch of the dendrogram corresponds to one of the measured samples labelled with the formulation and ageing time. Sample names are colour-coded according to the formulation with increasing colour transparency in correlation with ageing time (highest transparency for the most aged sample).

As a general observation, the unaged samples consistently fall within the same sub-cluster (cluster V). This suggests that the classification is primarily driven by the effects of ageing rather than by variations in the formulations analysed. Nonetheless, all PVAc (pure homopolymer) samples accumulate in the same sub-cluster with only a small distance to the unaged samples, indicating that the (physico-)chemical differences between these two groups are small as well and that PVAc does not exhibit significant changes due to ageing. Cluster VI, identified as ‘mostly 4000 h’, shows the greatest difference from the other samples, as indicated by its distinct placement in the dendrogram. This cluster mainly contains Pliodisp 7580 (PVAc + bis (2-ethlhexyl phthalate) (DEHP) + diethyl phthalate (DEP) + diisobutyl phthalate (DiBP) + di-*n*-octyl phthalate (DNOP) + 2-ehtylhexyl benzoate (2-EHB)), Mowilith LDM 1871 (PVAc + triethylene (TEG)), Inart Glue (PVAc + Diacetin + diethyleneglycol dibenzoate (DEGDB) + dipropyleneglycol dibenzoate (DPGDB)), and Pliodisp 7252 (Vac-co-VeoVa + Isonox 132^®^) samples, all aged for 4000 h (samples in triplicates). Hence, all of these samples show significant changes due to ageing. Moreover, Pliodisp 7252, aged for 2000 h, is assigned to the same sub-cluster, indicating that in contrast to all the other formulations, this sample already experiences significant ageing effects at an earlier point of exposure time (2000 h).

On the other hand, all aged Polidisp 1080 (PVAc + PVAl) samples accumulate in a separate sub-cluster (cluster I) that differs significantly from the clusters V (PVAc and unaged samples) and VI (mostly 4000 h). Therefore, it might be concluded that the (physico-)chemical properties of Polidisp 1080 change within the first 500 h of exposure, but remain considerably stable afterwards, even at 4000 h. Also, V2 Glue (E-co-Vac + diacetin + PVAl) aged for 1000 and 2000 h seems to exhibit similar features as aged Polidisp 1080, as they accumulate in the same sub-cluster (cluster I). However, in contrast to Polidisp 1080, V2 Glue aged for 4000 h shows more distinct changes (cluster VI).

The formulations Mowilith LDM 1871, Pliodisp 7580, and Pliodisp 7252 indicate similar ageing behaviour when aged for 500–1000 h (cluster III). Aged for 2000 h, Mowilith LDM 1871 and Pliodisp 7580 accumulate in another, even smaller sub-branch (cluster II), whereas for Pliodisp 7252, an accelerated ageing effect might be indicated (cluster VI).

The bi-plot in Figure 4 highlights some results of the multivariate analysis of selected FTIR-ATR bands and respective pH, CA, b* value, PD and M_W_ data. As already observed in the dendrogram, Mowilith LDM 1871, Pliodisp 7580, and Pliodisp 7252 show similar ageing behaviour. In addition, the bi-plot indicates that this clustering is strongly influenced by the FTIR bands located at 1172, 1151, and 1191 cm^−1^, all assigned to C-O stretching vibrations, whereas the bands in the CH-str. region have an opposite effect. This can also be seen in Figure 2, where these three formulations show decreasing band intensities in the CH str. region around 2900 cm^−1^. However, the bands in the fingerprint region (1172, 1151, and 1191 cm^−1^) slightly increase, showing the most dramatic increase for Pliodisp 7252. As already discussed above, different ageing behaviour is highlighted for Polidisp 1080, especially when compared to Mowilith LDM 1871, Pliodisp 7580, and Pliodisp 7252. This is indicated by the clustering of the scores in the bi-plot, with some changes due to ageing, strongly influenced by FTIR bands at 1434, 1372, 945, and 796 cm^−1^, all of which can be assigned to stretching or deformation vibrations of C-H bonds. The clustering of V2 Glue is strongly influenced by changes in contact angle and FTIR bands located in the CH str. region (around 2900 cm^−1^). Inart Glue also exhibits distinct changes due to ageing, being mostly affected by the PD and M_W_ parameters, which are both increasing, while the C-O stretch vibration at 1276 cm^−1^ decreases.

**Figure 5:**
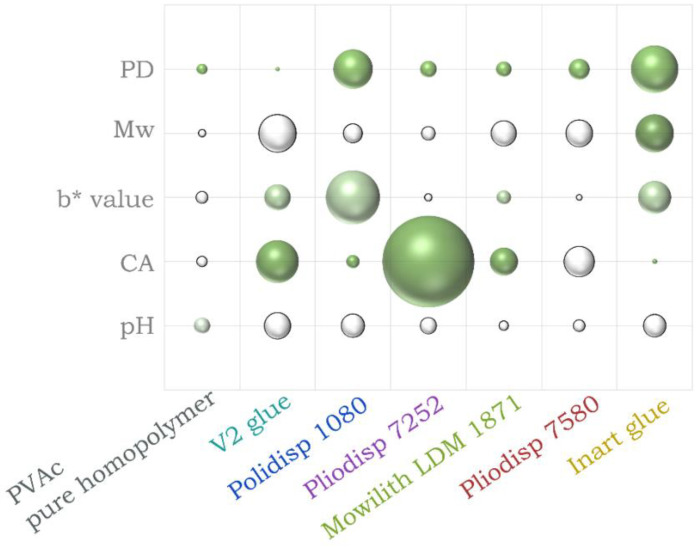
Percentage difference between aged and unaged samples for pH, contact angle (CA) and colour (b* value) measurements, molecular weight (M_W_), and polydispersity (PD) measurements for all VAc-based emulsions from t = 0 to t = 4000 h. Negative values are depicted in white and positive in green.

**Figure 6:**
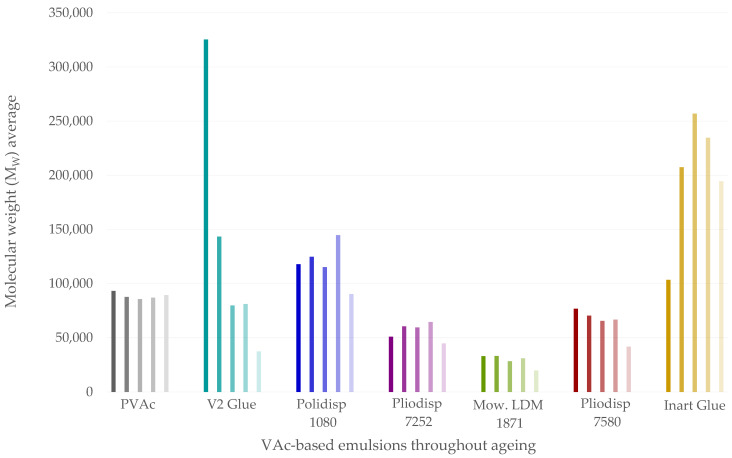
Graph exhibiting the molecular weight average for the different samples through ageing. The colour corresponds to formulation types and its transparency to the ageing time, (higher transparency indicates longer ageing time).

The analysis of the dendrogram and bi-plot in the figures above can be even better understood by analysing the results in Figure 5, which summarises the pH, CA, colourimetry, M_W_ and PD results for all samples, and Figure 6, which depicts the M_W_ variation throughout the ageing process. Two extra tables are available in the Supplementary Materials, one to showcase images of the films throughout ageing (Table S1) and one that clarifies Figure 5 by showing the percentage variation in numbers (Table S2).

A more detailed analysis of these results in conjunction with the information in the dendrogram and bi-plots are given for each formulation hereafter.

### 3.1. PVAc Pure Homopolymer

A previous study [4] indicates PVAc (pure homopolymer) as photochemically stable at λ ≥ 300 nm. In Figure 3 and Figure 4, PVAc remains in the same subregion from t = 0 to t = 4000 h. Thus, our results follow previous research conclusions and emphasise the role of additives and co-monomers in the ageing of VAc-based emulsions. Indeed, this sample showed the lowest variation in pH, CA, b* value (Figure 5) and FTIR-ATR spectra (Figure 2). Molecular weight variations were also negligible (within experimental error) (Figure 6) further indicating the stability of this polymer when not plasticised or copolymerised.

### 3.2. PVAc Homopolymer with Additives

#### 3.2.1. Polidisp 1080 (PVAc + PVAl)

Polidisp 1080 shows distinct behaviour, clustering separately from both the PVAc homopolymer and other formulations (Figure 3 and Figure 4). Initially clustering with the homopolymer at t = 0, it shifts to a different cluster from t = 500 to t = 4000 h. This formulation exhibited the highest degree of yellowing (Figure 5) and a significant pH decrease, commonly linked to PVAc degradation [45]. While PVAl, detected as the only additive, is typically an emulsion stabiliser, these findings suggest it has a negative impact on the film’s long-term stability. PVAl, often added to improve water affinity and act as a plasticizer, lowers the glass transition temperature (Tg) and increases film flexibility. However, since Polidisp 1080 ages differently from other plasticized PVAc emulsions (e.g., with benzoates or phthalates), PVAl may behave uniquely compared to common plasticizers. Furthermore, results also suggest that surfactants used in emulsion polymerization may alter the ageing process of PVAc films, which differ from those produced by non-aqueous polymerization and may be more prone to ageing.

#### 3.2.2. Mowilith LDM 1871 (PVAc + TEG)

According to the dendrogram and bi-plot, this formulation shows similar behaviour to other plasticised formulations (both internally and externally) such as Pliodisp 7580 (PVAc +bis(2-ethylhexyl phthalate) (DEHP) + Diethyl phthalate (DEP) + diisobutyl phthalate (DiBP)+ di-*n*-octyl phthalate (DNOP) + 2-ethylhexyl benzoate (2-EHB) and Pliodisp 7252 (VAc-co-VeoVa + Isonox 132^®^), which may point to the lower stability of more flexible PVAc films. These samples seem equally influenced by changes in the bands at 1172 and 1191 cm^−1^ (C-O stretching), which are assigned to the polymer. Figure 5 shows that M_W_ and PD measurements suffered a slight change, as did the CA (except for the Pliodisp 7252, which increased significantly). This may indicate that the ageing is mainly influenced by the morphology of the polymer and its polarity.

#### 3.2.3. Pliodisp 7580 (PVAc + DEHP + DEP + DiBP + DNOP + 2-EHB)

As previously mentioned, Pliodisp 7580 shows a similar ageing behaviour as Mowilith LDM 1871 and Pliodisp 7252 at 500 and 1000 h. However, at 2000 h, Pliodisp 7580 and Mowilith LDM 1871 continue to follow similar ageing patterns, while Pliodisp 7252 ages more strongly. Pliodisp 7580 became more hydrophilic with ageing and showed a decrease in M_W_ (Figure 5), which points to chain scission. As Pliodisp 7580 is plasticised with a wide array of different plasticisers, this susceptibility towards deterioration might have been promoted by the amorphous morphology resulting from the large number of plasticisers and further influenced by their migration.

#### 3.2.4. Inart Glue (PVAc + Diacetin + DEGDB + DPGDB)

In Figure 3, Inart Glue forms a distinct cluster (IV) between t = 500 h and t = 2000 h, only aligning with other formulations in the subcluster VI at t = 4000 h. This formulation exhibits a clear trend driven by increases in M_W_ and PD (Figure 5), likely indicating side-chain reactions leading to branching and crosslinking [1,2]. Although plasticisers such as diacetin and benzoates, which reduce the polymer’s Tg, may contribute to these reactions, diacetin is also found in V2 Glue, which demonstrated chain scission. Despite both formulations containing diacetin, their behaviours diverged—Inart Glue showed signs of branching, while V2 Glue underwent chain scission. This suggests that diacetin could be an unstable plasticiser, inducing significant molecular, chemical, and physical changes. However, the exact degradation mechanism is still unclear and requires further research.

### 3.3. VAc Copolymers with Additives

#### 3.3.1. V2 Glue (E-co-VAc + Diacetin + PVAl)

Figure 3 depicts a unique ageing trend in V2 Glue, as at times 500, 1000, 2000, and 4000 h, it is found in different subclusters throughout the dendrogram. Nevertheless, up to 2000 h, it shows some similar behaviours with Polidisp 1080 (also with PVAl) which can also be observed in the bi-plot (Figure 4). However, at 4000 h, the corresponding scores are located on the opposite side of the bi-plot, which might be caused by the diacetin content. At this time, a clear influence from both the CH-str. region bands and the contact angle are indicated but with opposite effects. While CA increased with ageing time, there was a significant and rapid decrease in M_W_, which showed the highest initial value. Consequently, it showed the strongest rate of scissions per chain, and its high initial polydispersity indicates chains with high M_W_ variation (Table S3). It is worth mentioning that Vaidergorin et al. [46] have highlighted the importance of the initial M_W_ values and proved that PVAc films with high M_W_, when irradiated with UV light, tend to show a significant increase in the insoluble fraction, indicating a high degree of crosslinking. Considering that V2 Glue has a high initial M_W_, the possibility of crosslinking should not be excluded, especially when an increase in CA has also been detected. However, only a small decrease in the carbonyl band at 1730 cm^−1^ and of the C-O/C-C stretching at 1225 cm^−1^ (Figure 1), were recorded by infrared spectroscopy and were probably related to the plasticisers [4].

#### 3.3.2. Pliodisp 7252 (VAc-co-VeoVa + Isonox 132^®^)

This is the only formulation copolymerised with VeoVa, and according to the literature, VAc copolymers with VeoVa are often proposed as a good alternative to externally plasticised formulations. However, according to Figure 3 and Figure 4, this formulation showed an early ageing pattern as at 2000 h it clustered with the 4000 h aged formulations (cluster VI). This may indicate a faster ageing rate, which was not expected. VeoVa has been previously reported as an additive of good stability, being advertised for its high water repellence and for greatly improving “hydrolytic stability, adhesion, water, and UV resistance” [47,48]. In fact, regarding the long-term solubility of VAc-co-VeoVa emulsions, a study found that water absorption of the films was reduced with the increasing addition of VeoVa [49]. Even though the increase in hydrophobicity was also observed in this study (Figure 5), this does not translate into a higher stability upon ageing. This means that whilst a VAc-co-VeoVa emulsion might prove sufficient for industrial/commercial purposes, its use for the conservation of works of art (e.g., as a binder or adhesive) might not be recommended as it is likely that it becomes less soluble with ageing, thus rendering it untreatable. Moreover, the addition of Isonox 132^®^ did not appear to improve the stability in the long-term.

### 3.4. A Closer Look at M_W_

The molecular weight (soluble fraction) values did not show a consistent trend, as some samples exhibited an increase in molecular weight before it eventually decreased. This was the case for Polidisp 1080, Pliodisp 7252 and Inart Glue, as can be seen in Figure 6. PVAc has been known to simultaneously undergo chain scission and crosslinking under ultraviolet irradiation [50], although main chain scission has been reported as the predominant mechanism [4]. This dual behaviour suggests that branching or crosslinking may occur during the initial stages of irradiation before chain scission becomes predominant at around 4000 h. Furthermore, it should be stated that the eventual formation of a gel fraction was not evaluated, and therefore, the possibility that, at least for some formulations, crosslinking might prevail should not be discarded.

It is worth noting that V2 Glue had the highest starting M_W_, more than three times the starting M_W_ of pure PVAc. Polymers with higher M_W_ are generally expected to exhibit superior long-term stability due to their longer chains and greater entanglements [5], which confer better mechanical strength, as well as enhanced thermal and chemical resistance. However, this was not the case for V2 Glue. One possible explanation is that the additives in this glue (diacetin and PVAl) and the co-monomer, ethylene (known to affect film stability if present in high quantities) could make the glue more susceptible to degradation, leading to an increased M_W_ loss.

For Inart Glue, the initial increase in M_W_ suggests branching, while the subsequent decrease indicates chain scission occurring at t = 4000 h. This behaviour could explain its distinct clustering pattern in the HCA. The branching likely resulted from side-chain reactions facilitated by the presence of plasticisers (e.g., benzoates and diacetin), which reduce the polymer’s Tg and make it more prone to such reactions. However, the subsequent chain scission led to a reduction in M_W_, altering its clustering behaviour.

Overall, these findings suggest that the M_W_ behaviour of PVAc-based emulsions is influenced by a combination of polymer morphology and the presence of additives. Further research is necessary to understand the underlying mechanisms driving these changes and to develop formulations that offer improved stability and performance over time.

## 4. Conclusions

This study provides novel insights into the ageing behaviour of VAc-based formulations by demonstrating how the addition of co-monomers and additives, such as plasticisers, significantly impacts emulsion stability. While PVAc as a pure homopolymer proved to be stable throughout ageing, more complex emulsion formulations degraded following different pathways, often becoming more acidic and exhibiting increased yellowing, particularly in V2 Glue, Polidisp 1080, and Inart Glue. Formulations containing well-regarded additives and co-monomers, like PVAl and VeoVa, did not exhibit superior long-term stability, underscoring the unpredictable influence of such additives under different conditions. Notably, even plasticised formulations demonstrated variable ageing behaviours, emphasising the need for further exploration of the role of plasticiser type and concentration. This work uniquely highlights that no single factor, such as plasticiser presence or polymer molecular weight, consistently determine the stability of these emulsions. The study’s use of a multi-sensor approach, combining several analytical techniques, allowed for an extensive investigation of degradation behaviours. Multivariate analysis further enabled the identification of key variables driving these ageing processes, providing a holistic perspective on VAc-based emulsions’ degradation.

## Figures and Tables

**Table 1:** Compositional information of the VAc-based emulsions used in this study based on infrared spectroscopy and Py-GC/MS analysis [6]. Compounds in grey lettering are external plasticisers or stabilisers.

Commercial Name	Polymer Type	Composition													
		VAc	Ethylene	VeoVa	Diacetin	TEG	DEHP	DEP	DiBP	DNOP	2-EHB	DEGDB	DPGDB	PVAl	Isonox 132^®^
PVAc	Homo-	x													
V2 Glue ^1^	Co-	x	x		x										
Polidisp 1080 ^1^	Homo-	x												x	
Pliodisp 7252 ^1^	Co-	x		x											x
Mowilith LDM 1871 ^3,4^	Homo	x				x									
Pliodisp 7580 ^2,4^	Homo	x					x	x	x	x	x				
Inart Glue ^5^	Homo-	x			x							x	x		

VAc: vinyl acetate; VeoVa: vinyl ester of versatic acid; Diacetin: glycerol diacetate; TEG: triethylene glycol; DEHP: bis(2-ethyl hexyl phthalate; DEP: diethyl phthalate; DiBP: diisobutyl phthalate; DNOP: di-*n*-octyl phthalate; 2-EHB: 2-ethylhexyl benzoate; DEGDB: diethylene glycol dibenzoate; DPGDB: dipropyleneglycol dibenzoate; PVAl: Poly(vinyl alcohol); Isonox 132^®^: di-t-butyl-4-butylphenol (antioxidant). ^1^ Purchased from Papelaria Fernandes (Lisbon, Portugal); ^2^ received from Resiquímica-Chemical Resins (Sintra, Portugal); ^3^ received from Univar Solutions (Portuguese distributor of Celanese Corporation; Maia, Portugal); ^4^ according to the technical datasheets these emulsions were described as an aqueous copolymer dispersion based on vinyl acetate and ethylene (Mowilith LDM 1871) and an aqueous copolymer dispersion with no plasticisers, based on vinyl acetate and an acrylic acid ester (Pliodisp 7580); ^5^ purchased from Ponto das Artes (Lisbon, Portugal).

**Table 2:** Selected spectral bands including tentative band assignments [14]. Capital letters indicate bands located in the CH stretching region, whereas lowercase letters indicate bands in the spectral fingerprint region.

Label	Wavenumber/cm^−1^	Tentative Band Assignment	
A	2974	ν_as_ (CH_3_)	PVAc
B	2963	ν (CH)	VeoVa
C	2949	ν_as_ (CH_2_)	PVAl
D	2941	ν (CH_2_)	PVAl/VeoVa
E	2932	ν_as_ (CH_2_)	PVAc
F	2922	ν_as_ (CH_2_)	Ethylene
G	2876	ν (CH_2_)	VeoVa
H	2863	ν_s_ (CH_3_)	PVAc
I	2860	ν (CH)	DEHP
a	1738	ν (C=O)	Diacetin
b	1730	ν (C=O)	PVAc
c	1717	ν (C=O)	PVAc
d	1709	ν (C=O)	PVAc
e	1462	CH	DEHP
f	1434	δ (CH)	PVAc
g	1372	δ (CH_3_)	PVAc
h	1276	ν (C-O)	Diacetin
i	1262	ν (C-O)	not assigned
j	1230	ν (C-O)	PVAc
k	1225	ν (C-O)	PVAc
l	1191	ν (C-O)	PVAc
m	1172	ν (C-O)	PVAc
n	1151	ν (C-O)	VeoVa
o	1131	CH	DEHP
p	1112	ν (C-O)	PVAc
q	1098	ν (C-O)	Benzoates
r	1071	ν (C-O)	DPGDB/DEGDB
s	1044	ν (CH)	DEHP
t	1018	ν (C-O)	PVAc
u	959	ν (C-O)	Benzoates
v	945	δ (CH) ν (C-C-O)	PVAc
w	796	δ (CH)	PVAc
x	716	δ (C=O)	DPGDB/DEGDB

PVAc—refers to poly(vinyl acetate), PVAl—poly(vinyl alcohol), DPGDB—dipropylene glycol dibenzoate, DEGDB—diethylene glycol dibenzoate, DEHP—Di(2-ethylhexyl) phthalate and VeoVa: vinyl ester of versatic acid; s—strong; m—medium; w—weak; vw—very weak; br—broad; sld—shoulder; ν—stretching vibration; δ—bending vibration.

## Data Availability

The original contributions presented in the study are included in the article/Supplementary Material, further inquiries can be directed to the corresponding authors.

## Supplementary Materials

The following supporting information can be downloaded at: https://www.mdpi.com/article/10.3390/polym16192841/s1, Figure S1: Score plots of all combinations of PCs 1–5 including the distribution of samples within each PC; Figure S2: Loadings of PCs 1–5; Table S1: Images of the films of glass slides during the ageing experiment showing the physical changes, namely yellowing (Polidisp 1080, V2 Glue and Inart Glue) and brittleness (PVAc, Pliodisp 7252 and Pliodisp 7580); Table S2: Percentage variation for pH, contact angle (CA) and colour (*b** value) measurements, molecular weight (M_W_), and polydispersity (PD) measurements for all VAc-based emulsions from t = 0 to t = 4000 h (V4000 h-V0 h)/(V 0 h × 100); Table S3: Number-average molecular weight (Mn), weight-average molecular weight (M_W_) and Polydispersity (PD) for samples over irradiation time.
