# Supplementary material for: The Role of Formulations in the Ageing Process of Vinyl Acetate Based Emulsion Films: A Multivariate Approach
Source: Polymers (Basel). 2024 Oct 8;16(19):2841. doi: 10.3390/polym16192841 (PMC11478713; doi:10.3390/polym16192841)
Supplement: Supplementary file 1 [file polymers-16-02841-s001.zip › polymers-3214370-supplementary.pdf]

polymers

MDPI

Article

The Role of Formulations in the Ageing Process of Vinyl Acetate Emulsions: A Comprehensive Multivariate Study

Carolina Viana [1], Karin Wieland [2,*], Susana França de Sá [1], Eva Mariasole Angelin [3], Valentina Pintus [4, 5] and Joana Lia Ferreira [6,*]

[1] LAQV-REQUIMTE, Department of Conservation and Restoration, NOVA School of Science and Technology, Universidade NOVA de Lisboa, 2829-516 Caparica, Portugal; c.viana@campus.fct.unl.pt (C.V); susana.sa@fct.unl.pt (S.F.d.S)
[2] Competence Center CHASE GmbH, Ghegastrasse 3, 1030 Vienna, Austria; karin.wieland@chasecenter.at
[3] Chair of Conservation-Restoration, Art Technology and Conservation Science, Technical University of Munich, Oettingenstr. 15, 80538, Munich, Germany; eva.angelin@tum.de
[4] Institute of Science and Technology in Art, Academy of Fine Arts, Schillerplatz 3, A-1010 Vienna, Austria; v.pintus@akbild.ac.at
[5] Institute for Conservation-Restoration, Modern-Contemporary Art, Academy of Fine Arts Vienna, Schillerplatz 3, A-1010 Vienna, Austria; v.pintus@akbild.ac.at
[6] CIUHCT – Interuniversity Center for the History of Sciences and Technology, Department of Conservation and Restoration, NOVA School of Science and Technology, Universidade NOVA de Lisboa, 2829-516 Caparica, Portugal; jlaf@fct.unl.pt

* Correspondence: karin.wieland@chasecenter.at (K.W.); jlaf@fct.unl.pt (J.L.F)

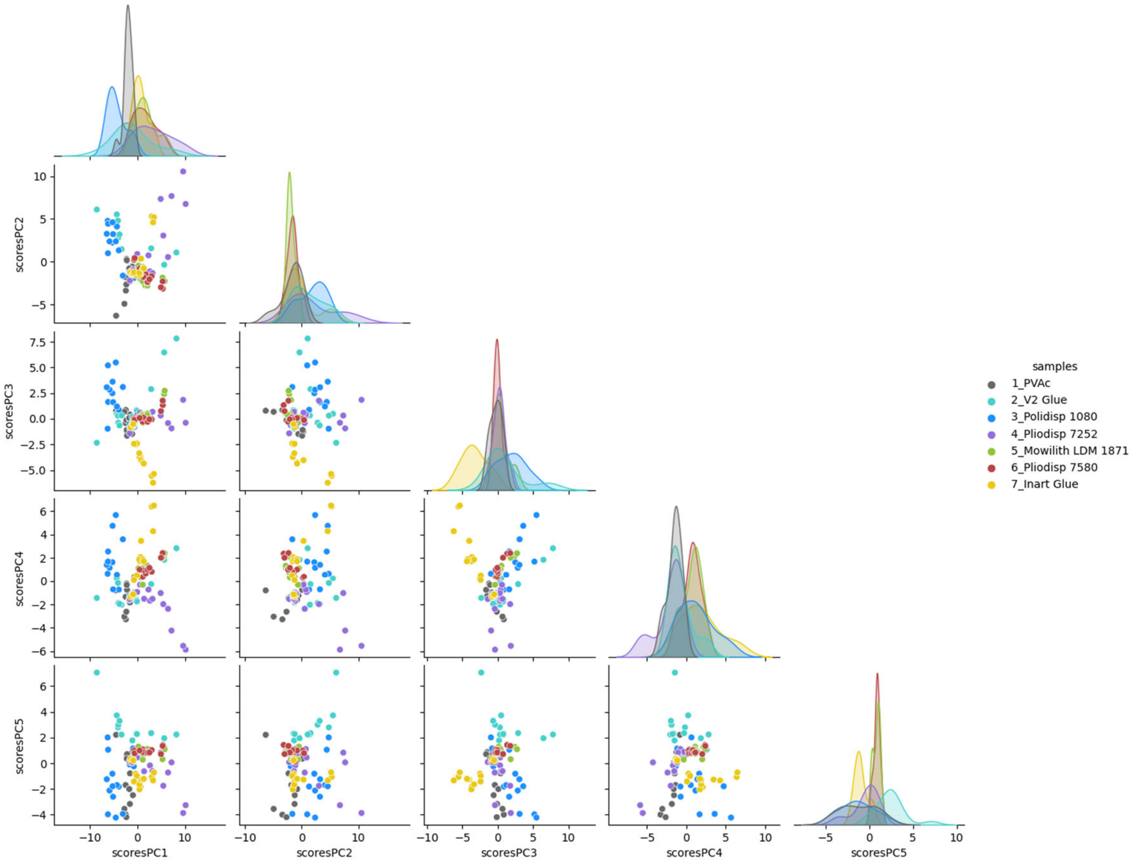

Figure S1. Score plots of all combinations of PCs 1-5 including the distribution of samples within each PC

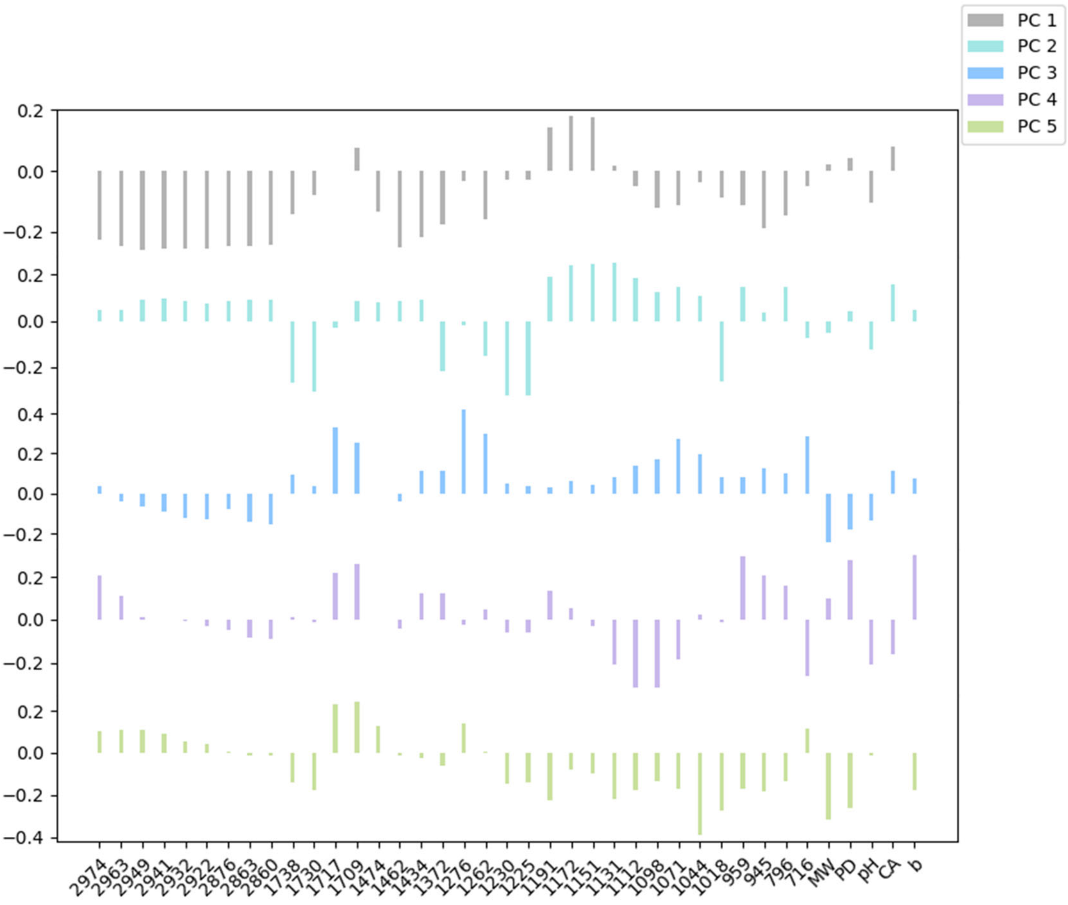

Figure S2. Loadings of PCs 1-5.

Table S1. Images of the films on glass slides during the ageing experiment showing the physical changes, namely yellowing (Polidisp 1080, V2 Glue and Inart Glue) and brittleness (PVAc, Pliodisp 7252 and Pliodisp 7580).

	t = 0h	t = 500h	t = 1000h	t = 2000h	t = 4000h
**PVAc Pure Homopolymer**	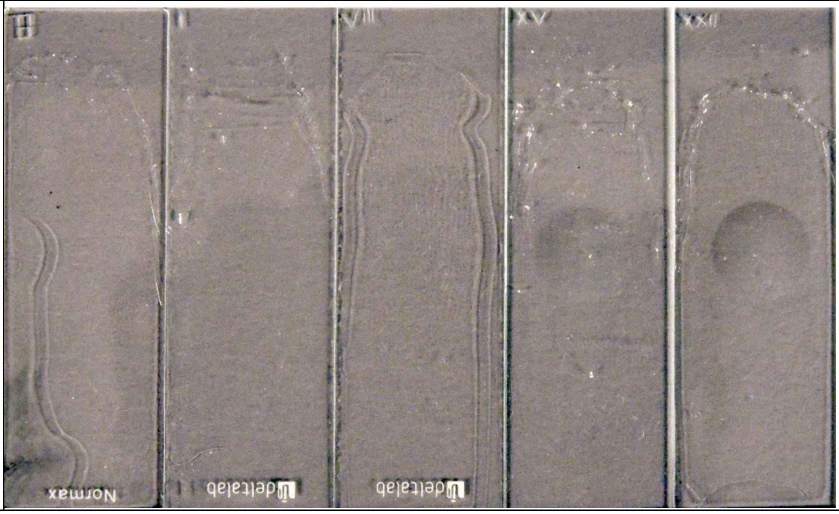				

Polidisp 1080	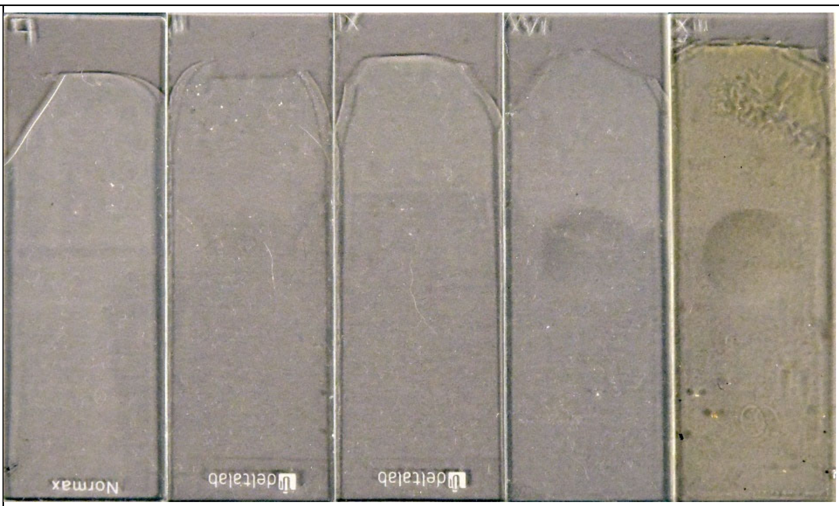
V2 Glue	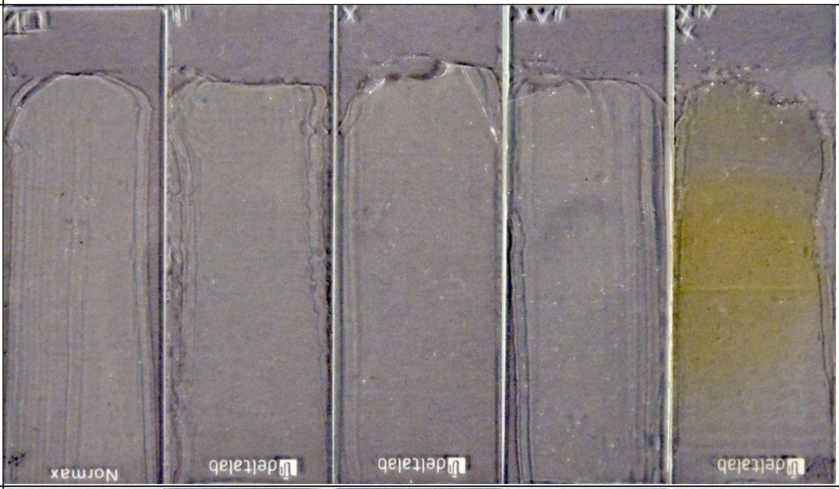
Pliodisp 7252	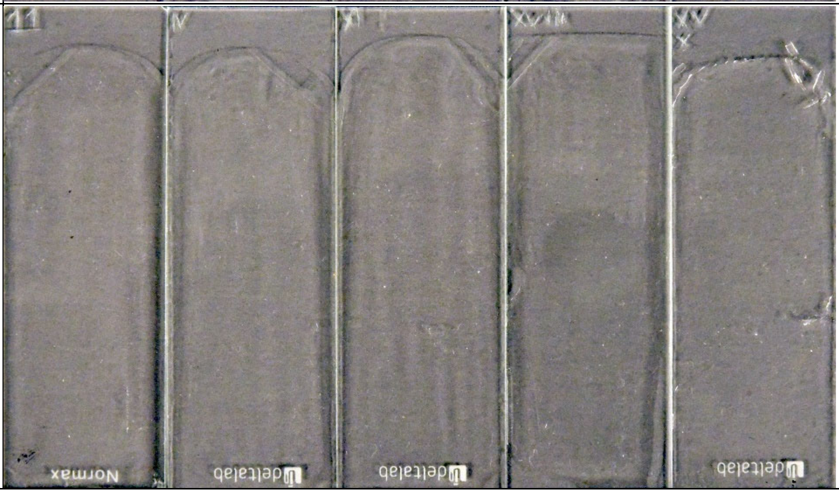

**Mowilith LDM 1871**	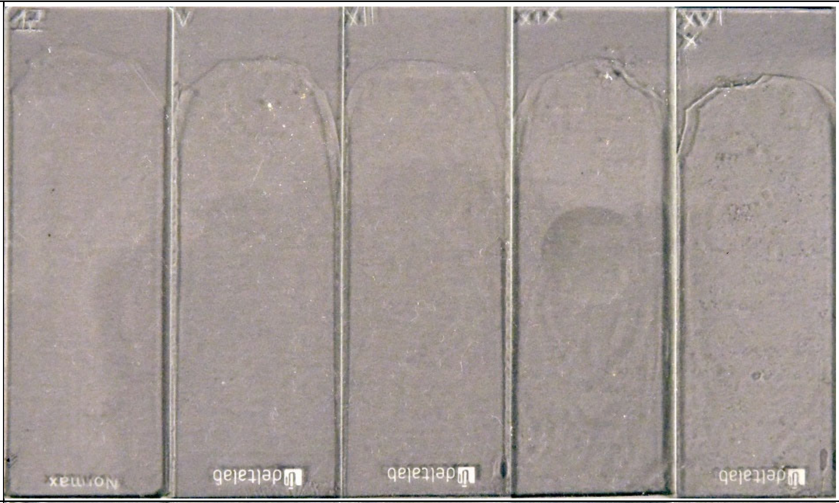
**Pliodisp 7580**	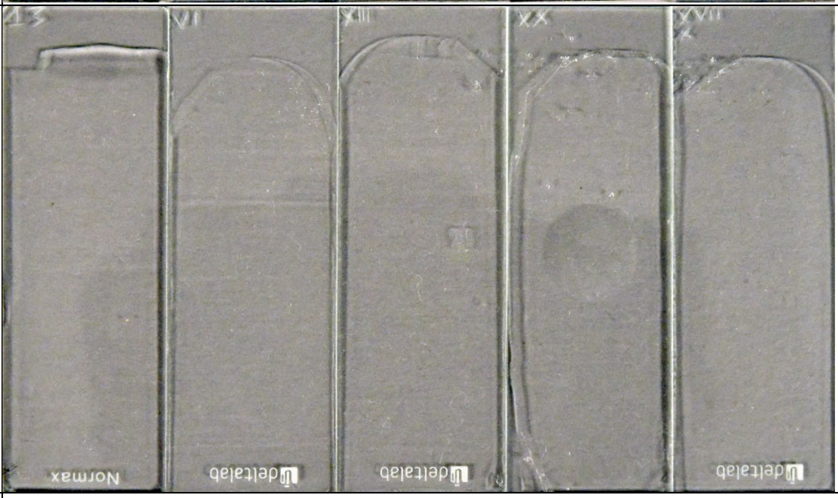
**Inart Glue**	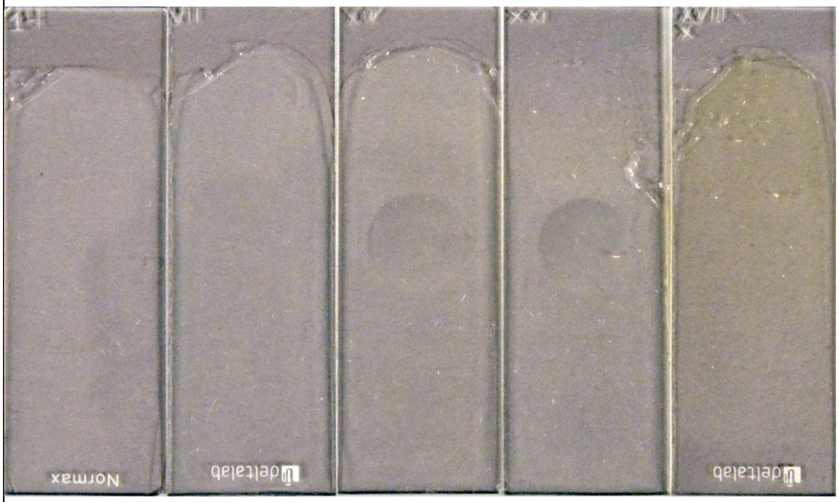

Table S2. Percentage variation for pH, contact angle (CA) and colour (b* value) measurements, molecular weight (MW), and polydispersity (PD) measurements for all VAc-based emulsions from t = 0 to t = 4000 hours ($(V_{4000h}-V_{0h})/V_{0h}*100$)) ($V_t$ = measured values after *t* hours of ageing)

	Variation between aged (4000 h) and unaged samples (%)				
	pH	CA	b* value	Mw	PD
PVAc pure homopolymer	15.94	-7.42	-9.43	-4.01	7.10
V2 glue	- 43.96	109.65	41.53	-88.51	1.06
Polidisp 1080	-34.68	10.76	177.00	-23.26	94.02
Pliodisp 7252	-17.34	509.00	-4.05	-12.40	16.66
Mowilith LDM 1871	-6.16	47.38	12.11	-40.36	13.92
Pliodisp 7580	-9.33	-58.30	-2.45	-45.68	26.14
Inart glue	-32.47	1.45	65.02	88.16	135.26

Table S3. Number-average molecular weight (M_n), weight-average molecular weight (M_W) and Polydispersity (PD) for samples over irradiation time.

		**M_n**	**M_W**	**PD**
**PVAc**	**0h**	47434	93079	1.97
	**500h**	44083	87569	1.99
	**1000h**	39801	85569	2.15
	**2000h**	43382	86941	2.01
	**4000h**	42439	89346	2.11
**V2 Glue**	**0h**	69061	325279	4.71
	**500h**	34125	143315	4.20
	**1000h**	21008	79678	3.80
	**2000h**	20010	81002	4.05
	**4000h**	7862	37374	4.76
**Polidisp 1080**	**0h**	47122	117745	2.51
	**500h**	41294	124656	3.04
	**1000h**	41250	115110	2.79
	**2000h**	36336	144665	3.99
	**4000h**	18598	90347	4.87
**Pliodisp 7252**	**0h**	25669	50917	1.98
	**500h**	24996	60371	2.42
	**1000h**	23415	59520	2.54
	**2000h**	23364	64393	2.76
	**4000h**	19318	44599	2.31
**Mowilith LDM 1871**	**0h**	9350	32896	3.52
	**500h**	10272	33143	3.23
	**1000h**	8675	28279	3.27
	**2000h**	8388	30854	3.68
	**4000h**	4902	19618	4.01

**Pliodisp 7580**	**0h**	31890	76802	2.41
	**500h**	22651	70266	3.11
	**1000h**	20253	65448	3.23
	**2000h**	18747	66642	3.59
	**4000h**	13739	41712	3.04
**INART Glue**	**0h**	49965	103353	2.07
	**500h**	45915	207454	4.52
	**1000h**	52714	256959	4.88
	**2000h**	45906	234566	5.11
	**4000h**	40086	194474	4.87

Disclaimer/Publisher's Note: The statements, opinions and data contained in all publications are solely those of the individual author(s) and contributor(s) and not of MDPI and/or the editor(s). MDPI and/or the editor(s) disclaim responsibility for any injury to people or property resulting from any ideas, methods, instructions or products referred to in the content.
